# Transcriptomic analysis reveals that RasGEF1b deletion alters basal and LPS-induced expression of genes involved in chemotaxis and cytokine responses in macrophages

**DOI:** 10.1038/s41598-023-47040-9

**Published:** 2023-11-10

**Authors:** Heliana B. Fernandes, Isadora Mafra de Oliveira, Thomas S. Postler, Sérgio Q. Lima, Cícera A. C. Santos, Michaelle S. Oliveira, Felipe B. Leão, Sankar Ghosh, Maria C. Souza, Warrison Andrade, Aristóbolo M. Silva

**Affiliations:** 1 Laboratory of Inflammatory Genes, Instituto de Ciências Biológicas, Universidade Federal de Minas Gerais (UFMG), Belo Horizonte, MG 31270-901 Brazil; 2 Department of Microbiology & Immunology, Vagelos College of Physicians & Surgeons, Columbia University Irving Medical Center, New York, NY USA; 3 Instituto Federal de Educação, Ciência e Tecnologia de Rondônia (IFRO), Guajará-Mirim, RO Brazil; 4 Present Address: Faculdade de Medicina de Ribeirão Preto, Av. Bandeirantes, 3900 - Campus da USP, Ribeirão Preto, SP 14049-900 Brazil; 5 Present Address: Design and Development Laboratory, International AIDS Vaccine Initiative, Brooklyn, NY USA

**Keywords:** Gene expression analysis, Monocytes and macrophages, Gene ontology, Gene regulation, Gene expression, Transcriptomics, RNAi, Gene expression profiling, Gene targeting

## Abstract

Ras guanine nucleotide exchange factor member 1b (RasGEF1b) of the RasGEF/CDC25 domain-containing family is preferentially expressed by macrophages. However, information is lacking about its role in macrophage function. In this study, we generated mice with ubiquitous deletion of *Rasgef1b* and used RNA-seq-based transcriptomics to compare the global gene expression in wild-type and knock-out primary bone-marrow-derived macrophages under basal conditions and after lipopolysaccharide (LPS) treatment. Transcriptional filtering identified several genes with significantly different transcript levels between wild-type and knock-out macrophages. In total, 49 and 37 differentially expressed genes were identified at baseline and in LPS-activated macrophages, respectively. Distinct biological processes were significantly linked to down-regulated genes at the basal condition only, and largely included chemotaxis, response to cytokines, and positive regulation of GTPase activity. Importantly, validation by RT-qPCR revealed that the expression of genes identified as down-regulated after LPS stimulation was also decreased in the knock-out cells under basal conditions. We used a luciferase-based reporter assay to showcase the capability of RasGEF1b in activating the *Serpinb2* promoter. Notably, knockdown of RasGEF1b in RAW264.7 macrophages resulted in impaired transcriptional activation of the *Serpinb2* promoter, both in constitutive and LPS-stimulated conditions. This study provides a small collection of genes that shows relative expression changes effected by the absence of RasGEF1b in macrophages. Thus, we present the first evidence that RasGEF1b mediates the regulation of both steady-state and signal-dependent expression of genes and propose that this GEF plays a role in the maintenance of the basal transcriptional level in macrophages.

## Introduction

Ras guanine nucleotide exchange factors (RasGEFs) are a specialized group of cytosolic proteins that replace bound guanosine diphosphate (GDP) with guanosine triphosphate (GTP) in GTPases, causing them to become active^1^. These proteins have been classified according to the presence of two domains: an amino-terminal Ras exchange motif (REM) structural domain and a CDC25 homology catalytic domain (CDC25-HD), also called RasGEF domain, based on its homology to the protein Cdc25 from *S. cerevisiae*, the first RasGEF to be identified^2^. In humans and mice, the RasGEF family is represented by 30 and 27 members^3^, respectively, being categorized into four subfamilies: Sos, RasGRF, RasGRP, and CNRasGEF^4^, which exclusively regulate the small Ras-like GTPases Ras, Rap, and Ral^3^.

RasGEFs have been implicated in the regulation of several cellular processes such as cell proliferation, survival, differentiation, actin organization, vesicular trafficking, and gene expression^1,5^. The cellular functionality of the specific RasGEF/Ras modules is primarily regulated by the tissue-specific expression pattern of its components^6,7^. RasGEF domain-containing proteins can regulate multiple GTPases, and individual GTPases can be regulated by more than one GEF, creating a complex biological interplay of tremendous functional diversity^4,8,9^. This complexity is compounded by the large number of poorly characterized GEFs, making tissue-specific studies imperative.

RasGEF domain-containing member 1b (RasGEF1b) belongs to a restricted group that does not share any particular domain with the related family members 1a and 1c–beyond their shared structural REM and catalytic CDC25-HD domains–^10^, and exhibits specific GEF activity for Rap2 GTPase, but not for Rap1 or other members of the Ras subfamily^11,12^. RasGEF1b expression is detectable in unstimulated macrophages, and it can be further increased by innate immune stimuli triggered through Toll-like receptor (TLR) activation requiring the NF-κB transcription factor for maximal induction^13,14^. In mice, its expression appears to be more abundant in the brain and spleen, and it is increased following protozoan infections^13^. It was reported that in addition to the linear mRNA, a circular RNA (circRNA) of RasGEF1b generated through back-splicing of the exons 2 and 4 is detectable and induced by LPS in murine macrophages^15^.

Despite biochemical and expression studies, little is known about the cellular function of RasGEF1b, especially in macrophages where its expression is particularly high. Based upon this observation, we hypothesized that RasGEF1b may play a role in macrophages under resting or inflammatory conditions. Here, we used the Cre-*loxP* technique to generate mice with a ubiquitous deletion of *Rasgef1b* to obtain primary bone-marrow-derived macrophages (BMDMs), which we then used to further examine and compare gene expression in primary resting or LPS-activated wildtype (WT) and knock-out (KO) macrophages with high-resolution transcriptomics. RNA-seq analysis revealed that *Rasgef1b* deletion disturbs both the baseline and LPS-induced expression of a small subset of genes involved in chemotaxis, cytokine responses, and positive regulation of GTPases.

## Materials and methods

### Rasgef1b gene targeting

To generate mice with a floxed *Rasgef1b* allele for conditional deletion, exons 7 and 8 were chosen for targeting because Cre-mediated recombination on these exons causes a frameshift resulting in a stop codon early in exon 9, resulting in truncation of the entire C-terminus – which comprises the Cdc25/Ras homology catalytic RasGEF domain. A *loxP* site was introduced upstream of exon 7, and an *Frt-Neo*^*R*^*-Frt-loxP* cassette was inserted downstream of exon 8 by sequential rounds of recombineering^16–18^. The resulting targeting vector was electroporated into C57BL/6 background embryonic stem (ES) cells and selection was performed with G418 (Invitrogen). To confirm targeting of the *Rasgef1b* allele for the homologous recombination event, genomic DNA extracted from ES G418-resistant cells was digested with *Afl*II and *Eco*RI and then hybridized with individual^32^P-labeled 5′ and 3′ probes for Southern-blot analysis. Bands of 4.7 kb and 3.8 kb sizes were expected for 5′ and 3’probes in the targeted allele, respectively. Targeted ES-cell clones were microinjected into MF1 blastocysts for generation of chimeric mice, which were then crossed with C57BL/6 mice to obtain animals containing the *Rasgef1b*^flox/Frt/Neo^ allele in the germline. The neomycin resistance gene was then removed by breeding the latter with mice carrying ‘‘Flip’’ recombinase to generate the *Rasgef1b*^+/flox/Frt-^. These procedures were performed by the Gene Targeted Mouse Service Core at the University of Cincinnati, USA. Homozygous *Rasgef1b*^flox/flox^ (designated *Rasgef1b*^fl/fl^ for short) and Cre-mediated knock-out mice were generated at the animal facility at the University of Massachusetts Medical School (UMMS, USA). To generate mice with disruption of *Rasgef1b* in all tissues, female *Rasgef1b*^f/f^ mice were first crossed with male B6.C-Tg(CMV-cre)1Cgn/J mice^19^ (CMV-Cre, The Jackson Laboratory, stock no. 006054) that express the Cre recombinase, an X-linked transgene, under the transcriptional control of the human CMV minimal promoter. Adult heterozygous mice were later intercrossed to eventually generate mice with a global deletion of *Rasgef1b*. All mice used in this study were maintained on a C57BL/6 background. Mice were maintained under specific pathogen-free conditions. All procedures used in this study were approved by the Institutional Animal Care and Use Committee (UMMS, IACUC nos. 1817-09, A-2057, A-2073) and the Ethic Committee on Animal Use (CEUA) of Federal University of Minas Gerais and institutionally under protocol number 047/2017 and 64/2019. This study was performed in accordance with the ARRIVE guidelines. All methods were carried out in accordance with relevant guidelines and regulations.

### PCR genotyping

*Rasgef1b*^fl/fl^ mice genotyping was routinely carried out by PCR with the primers P3: 5′-GTATTTGGGCAGCATGTATGTC-3′ and P4: 5′-AGGTGTGTAAATGGCCACTGAG-3′. The expected amplicon sizes are 320 bp and 454 bp for *Rasgef1b* wild-type (WT) and conditional floxed (fl/fl) alleles, respectively. Alternatively, mice were genotyped for the (5′) proximal *loxP* site by using the primers P1: 5′CCAAGAGCAGAGTGAAACATGC-3′ and P2: 5′AGAGTGAGCTACATAAGAAAGC-3′ to generate amplicons with the size of 108 bp and 152 bp for WT and targeted, respectively. Genotyping of mice with conditional gene deletion after Cre recombination was performed with the primers P1 and P4 to generate expected amplicons of 1466 bp for the WT and 317 bp for the knock-out. Twenty microliter of PCR reactions containing genomic DNA (20–50 ng) extracted from the tails, 20 pmol of each primer, dNTP mix 200 μM, standard 1X PCR buffer, 1U Taq DNA polymerase (Phoneutria Biotecnologia, Brazil) were run at 94 °C/2 min, 30 cycles at 94 °C/30 s, 55 °C/30 s, 72 °C/1 min, and final extension at 72 °C/3 min (MyCycler, BioRad). PCR products were separated on a 1.5% agarose gel followed by staining with ethidium bromide. Recombinase transgene alleles were confirmed with the following primers sequences: CMV-cre, Cre1: 5′CATCGCCATCTTCCAGCAG-3′and Cre2: 5′CAATTTACTGACCGTACAC-3′.

### Isolation of bone-marrow-derived macrophages

Eight- to 12-week-old mice were used to isolate bone-marrow cells from the femur and tibia bones to make cultures of bone-marrow-macrophages (BMDMs). WT C57BL/6 mice were provided by the animal facility Centro de Bioterismo (CEBIO), UFMG, Brazil. Mice were euthanized following ketamine/xylazine anesthesia in accordance with institutional and national guidelines for the care and use of laboratory animals. Briefly, bone-marrow cells were plated on non-treated 10-cm plates and cultured in RPMI 1640 medium supplemented with 20% heat-inactivated FBS (Gibco Invitrogen, USA), 25 mM HEPES, 2 mM glutamine, 100 U/ml penicillin, 100 μg/ml streptomycin (Gibco, Invitrogen), 50 μM 2-mercaptoethanol as well as 30% (v/v) L929 cell-conditioned medium. Culture medium was replaced by fresh medium every 2–3 days. Adherent macrophage monolayers were obtained within 7–9 days; these cells were > 95% positive for F4/80 and CD11b antibody staining as determined by flow cytometric analysis, identifying them as mature macrophages (data not shown). BMDMs were then harvested by gently resuspending the cells from the dishes using cold PBS and seeded onto cell cultures plates for the experiments.

### Quantitative RT-PCR (RT-qPCR)

Total RNA from untreated and LPS-treated WT and RasGEF1b-cKO BMDMs was extracted using TRIzol® (Life Technologies) according to the manufacturer’s instructions. Total RNA was reverse transcribed using MMLV reverse transcriptase (Life Technologies). Quantitative PCR was performed using 2X Sso SYBR Green mix (BioRad) with a CFX96 Touch™-Real-Time detection system (BioRad). Oligonucleotide primer sequences were either obtained from the PrimerBank public database^20,21^ or designed through the freely available Primer3 website. Oligonucleotide synthesis was carried out by Integrated DNA Technologies (IDT Inc., USA) or GenOne Biotech (Rio de Janeiro, RJ, Brazil). Primer sequences used in the expression analyses in this study are listed in Supplementary Table S5. Expression data obtained in the quantitative RT-qPCR analyses of gene expression levels were normalized to those of the reference gene *Rpl32* (Large Ribosomal Subunit Protein L32).

### Generation of stable RasGEF1b-shRNA expressing RAW264.7 cells

Lentiviral pLKO.1 vector carrying short hairpin (shRNA) targeting mouse RasGEF1b mRNA transcript was obtained from Sigma. Non-targeting pLKO.1 shRNA vector carrying the shRNA targeting human RASGEF1B mRNA transcript was used as a control. Lentivirus stocks were prepared as vesicular stomatitis virus G (VSV-G) pseudotypes by transfecting HEK293T cells with endotoxin free midi-preps of pLKO.1 vectors, psPAX2 and pMD2.G at a ratio of 4:3:1 using polyethyleneimine (PEI). RAW264.7 cells (1 × 10^6^) were transduced in a 6-well dish with 1.5 ml of the virus and selected in medium containing 5.0 μg/ml puromycin three days later.

### Plasmids and promoter transcriptional assay

The murine *Serpinb2* region containing the proximal promoter (− 539/ + 92) cloned into the pGL3 reporter vector (pGLmP-539) was kindly provided by Dr. Toni M. Antalis (University of Maryland School of Medicine, MD, USA)^22^. RasGEF1b (NM_145839) was cloned into pcDNA3.1 + -DYK to be expressed in mammalian cells as a tagged protein with an N-terminal DYKDDDDK (FLAG®) tag (GenScript Biotech, USA). pFLAG-CMV4 was used as empty vector. The pRL-TK plasmid (Renilla luciferase) was used for normalization. Endotoxin free plasmids midi-preps were transfected using polyethylenimine “MAX” MW 40,000 (Polysciences, USA). HEK293 cells (2.5 × 10^5^ cells/well) or shRNA control and shRNA RasGEF1b-expressing RAW264.7 cells (2 × 10^5^ cells/well) were seeded in 24-well plates and transfected with 400 ng of pGLmP-539 and 100 ng of pRL-TK. For co-transfection experiments, HEK293 cells were also transfected with 3 μg of each expression plasmid. Cell lysates were harvested 48 h post-transfection or LPS treatment as indicated in figures. After discarding the medium by aspiration, cells were lysed with 200 μl of Passive Lysis Buffer (Promega). Ten microliters of cell lysate were assayed using the Dual-Luciferase Reporter Assay System (Promega) following the manufacturer's recommendations, and luminescence was measured using a Luminoskan microplate luminometer (ThermoScientific). Promoter activity was assessed by normalizing the ratio of Firefly:Renilla luciferase, and the results were presented as relative luciferase activity. To account for potential effects of experimental conditions on Renilla luciferase activity in HEK293 cells, the data were also normalized to cellular protein concentration. Protein concentration was determined using the Bio-Rad protein assay reagent to ensure accurate measurement.

### RNA sequencing (RNA-seq) and data analysis

RNA-seq was performed with RNA obtained from a total of three biological replicates per condition and genotype. Briefly, wild-type and RasGEF1b-cKO BMDMs were plated at 2 × 10^5^ cells per well in 6-well tissue culture plates. Cells were then left untreated or treated with 100 ng/ml of lipopolysaccharide (LPS) from *Escherichia coli* O55:B5. After 4 h of incubation at 37 °C in an atmosphere of 5% CO_2_, the medium was discarded and the cells were washed in cold phosphate-buffered saline (PBS). Total RNA was extracted using TRIzol reagent following the manufacturer’s protocol (Thermo Fisher Scientific). RNA samples were resuspended in nuclease-free water and further analyzed for concentration and integrity by spectrophotometer (NanoDrop™ Lite, ThermoScientific) and Bioanalyzer 2100 (Agilent Technologies, Santa Clara, CA, USA), respectively. RIN (RNA Integrity Number) values for all samples were equal to or higher than 9.8 as measured by Bioanalyzer indicating high integrity of the RNA samples. Library construction and RNA sequencing were performed by Genewiz, LLC (South Plainfield, NJ, USA). Removal of ribosomal RNA was achieved by poly-A selection for mRNA and other polyadenylated RNA species. Libraries were built from three independent RNA samples obtained from WT and RasGEF1b-cKO BMDMs cultures as biological replicates per condition. The 12 cDNA libraries were prepared using the NEBNext Ultra II RNA Library Prep Kit for Illumina following the manufacturer’s instructions (NEB, Ipswich, MA, USA), and sequenced on an Illumina HiSeq sequencer in a paired-end 150-bp configuration. Raw sequence reads were aligned to the mouse genome (assembly GRCm38/mm10, annotation release 100) using Spliced Transcripts Alignment to a Reference (STAR) v2.7.4a^23^. For quality control, we used the Quality of RNA-Seq ToolSet (QoRTs) v1.3.6, and Integrative Genomics Viewer (IGV) v2.8.2 software^24,25^. Differential gene expression analyses were conducted using DESeq2 in the R software environment (v4.0.0 and higher) (51–53). Significant differentially expressed genes were defined as *p* < 0.05 after adjustment for false discovery rate and a fold change > 2 between the compared groups, as calculated by DESeq2^26–28^.

### Functional enrichment analysis and gene ontology

Functional analyses of differentially expressed genes identified in the knock-out macrophages were carried out using the Database for Annotation, Visualization and Integrated Discovery (DAVID)^29^ and compared with PANTHER^30^. For pathway enrichment analysis, we used the significantly down-regulated genes as input in the InnateDB (www.innatedb.com) analysis platform using Benjamini–Hochberg false discovery rate correction, adjusted *p*-value < 0.01 and hypergeometric distribution.

### Statistical analysis

For RNA-seq, comparison between genotypes and conditions were made between different samples of different BMDMs samples per mice and conditions (unpaired). Additional statistical analyses were performed using GraphPad Prism™ 9.0.2 software. Results are given as mean ± SEM or SD where indicated. Comparisons between two groups were done using unpaired t-test. Multiple comparisons were done with one-way analysis of variance (ANOVA). Statistical significance was determined as *p < 0.05; **p < 0.01.

## Results

### RasGEF1b is preferentially expressed in macrophages among members of the RasGEF1 subfamily

**Figure 1:**
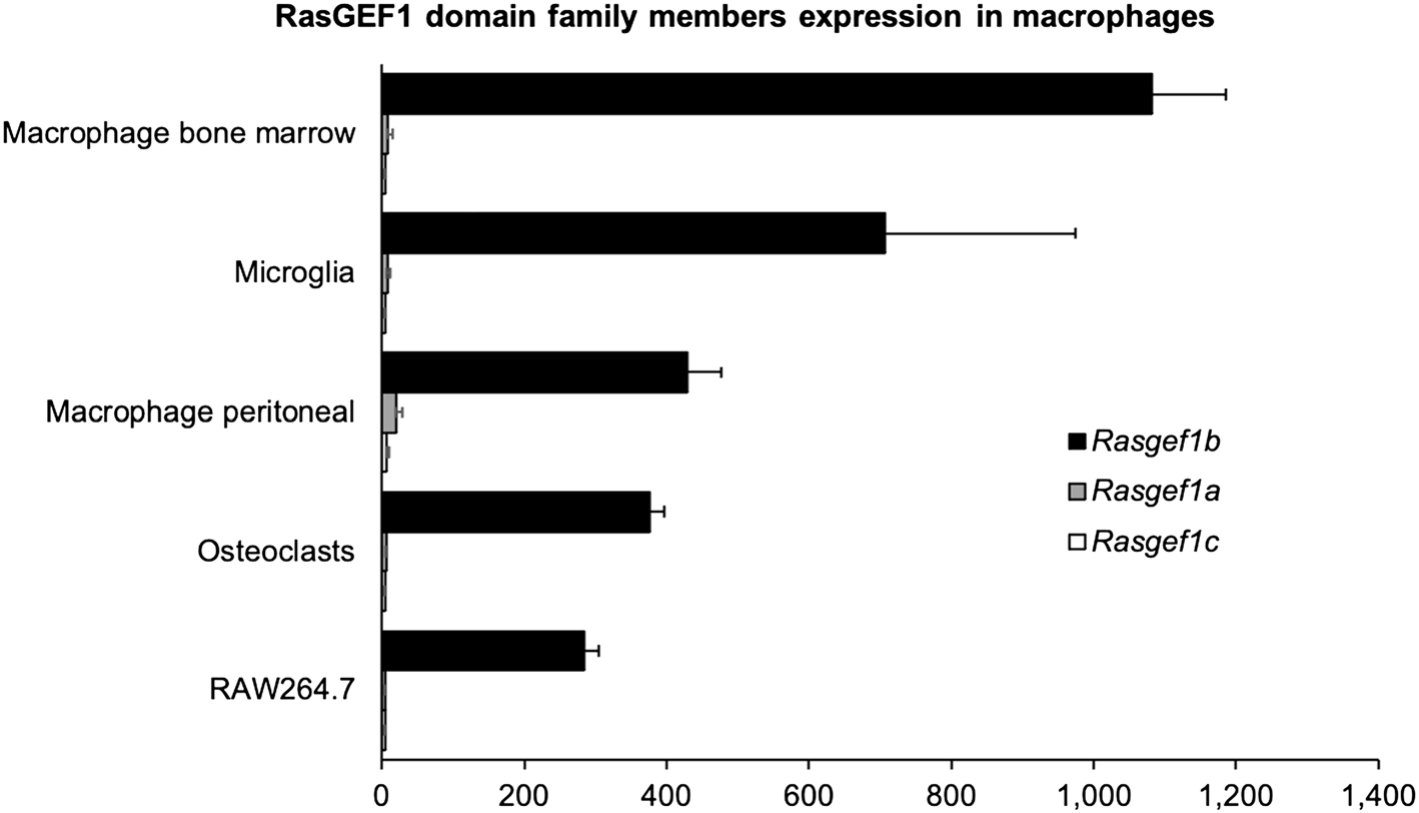
Preferential expression of RasGEF1b among RasGEF1 family members in macrophages. cDNA Expression level of RasGEF1 domain family members mouse macrophages from BioGPS database^31^.

We have previously shown that RasGEF1 domain family member 1b is found on murine macrophages and that its expression is induced upon Toll-like receptor (TLR) stimulation and regulated by NF-κB transcription factor^13,14^ To understand the function played by RasGEF1b in macrophage biology, we performed in silico analyses of the BioGPS database to compare its expression with the closest members RasGEF1a and RasGEF1c in mouse macrophage lineages^31^. We found that RasGEF1b exhibited the highest levels of expression in bone-marrow-derived macrophages, microglia, peritoneal macrophages, osteoclasts, and RAW264.7 cells, followed by RasGEF1a and RasGEF1c (Fig. 1, Supplementary Table S1). These results indicate that RasGEF1b expression is dominant over members of the RasGEF1 subfamily in macrophages.

### Disruption of the Rasgef1b gene in mice

**Figure 2:**
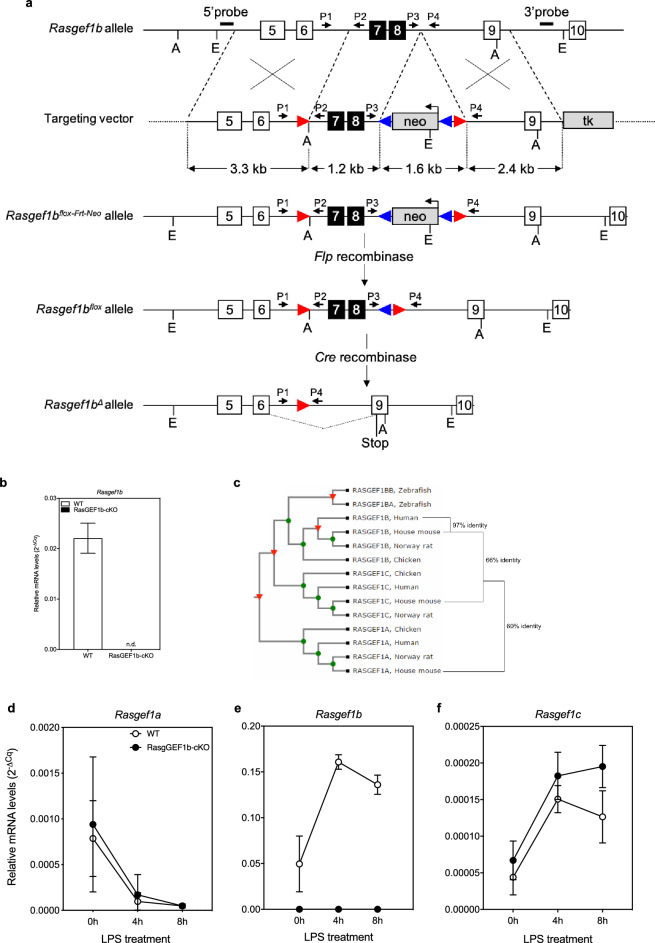
Gene targeting and generation of transgenic C57BL/6 *Rasgef1b*^fl/fl^ mice and conditional cre recombinase-mediated gene deletion. (**a**) Shown is a schematic of the strategy used for the generation of the floxed and conditional RasGEF1b knock-out mice. Illustrative maps are provided for the wild-type *Rasgef1b* allele, targeting vector, targeted allele, floxed *Rasgef1b* allele (flox) after Flp-mediated recombination, and the deleted *Rasgef1b* locus (Δ) in the targeted cells. The open or black boxes represent coding exons; the black boxes represent the targeted exons. Red and blue arrowheads indicate *loxP* and frt sites, respectively; A = *Afl*II; E = *Eco*RI. Approximate position of primers (P1–P4) used for genotyping are indicated by the horizontal arrows above line. The paired *loxP* sites in *Rasgef1b*^fl/fl^ alelles are located in introns 6 and 8. Cre recombinase activity on *Rasgef1b*^fl/fl^ alleles will result in the deletion of exons 7 and 8, causing both a frameshift and stop codon at exon 9 of the *Rasgef1b* upon Cre-mediated recombination in the target tissue or cells; this will eliminate the expression of the carboxy-terminal region of RasGEF1b that contains the catalytic domain for guanine nucleotide exchange. (**b**) PCR analysis of tail genomic DNA in 1.5% agarose gel used for routine genotyping. The PCR reactions contained the primers P3 and P4 (refer to panel (**a**), and *Material and Methods*) for diagnosing the flox allele. (**b**) RT-qPCR analysis of RasGEF1b mRNA levels in BMDMs differentiated from bone-marrow cells taken from wild-type (WT) and RasGEF1b-cKO (KO) mice. Data are represented as mean ± SEM (n = 3 per group). n.d., *not detected*. (**c**) Phylogenetic tree showing the similarity among a RasGEF1 proteins. The amino-acid sequences of RasGEF1b members of the organisms were aligned using the Clustal W program to generate the dendrogram. (**d**–**f**) RT-qPCR analysis of mRNA levels of RasGEF1 members in wild-type (WT) or RasGEF1b-cKO BMDMs that were left untreated or treated with LPS (100 ng/ml) for the time intervals indicated in the graphs.

To inspect the function of RasGEF1b in primary BMDMs, we developed a *Rasgef1b* knock-out mouse. The murine *Rasgef1b* gene consists of 14 exons and spans about 35 kb on the reverse strand of mouse chromosome 5 (NCBI ID: NC_000071.6, Ensembl accession: ENSMUST00000031276.14, UCSC accession: uc008ygl.1). Disruption of *Rasgef1b* in mice was achieved using standard homologous targeting and Cre recombinase-mediated strategies (as described in the *Materials and Methods* section; Fig. 2a). First, a targeting vector was created that contained a neomycin-resistance gene (neo), flanked by Frt sites, inserted between two *loxP* sites flanking exons 7 and 8, which encode part of the catalytic domain. Following electroporation and G418-mediated positive selection of embryonic stem (ES) cells, Southern blot analysis of four ES cell clones demonstrated successful targeting of the *Rasgef1b* allele (Supplementary Fig.S1a, S4). After breeding with Flipase-expressing mice to delete the neomycin cassette, heterozygous mice were generated and then intercrossed to generate homozygous *Rasgef1b*^fl/fl^ animals. To obtain complete *Rasgef1b*-null mice, *Rasgef1b*^fl/fl^ animals were crossed with cytomegalovirus (CMV)-Cre mice to obtain global *Rasgef1b* heterozygous mice, which were intercrossed to generate mice with deletion of *Rasgef1b* in all tissues, including germ cells. The knock-out mice (RasGEF1b-cKO) are fertile, present no apparent gross abnormalities, and are not visibly different from C57BL/6 wild-type mice. The absence of RasGEF1b in RasGEF1b knock-out animals was confirmed by assessing mRNA levels in differentiated BMDMs, fresh bone-marrow cells, spleen, lungs, liver, and heart (Fig. 2b and Supplementary Figs. S1b, S1c). Importantly, no compensatory expression by the closely related RasGEF1a and RasGEF1c was observed in the knock-out macrophages (Fig. 2c–f).

### RNA-seq, quality control, and read mapping

To determine the overall effect of *Rasgef1b* deletion on gene expression in macrophages, we performed RNA-seq on total RNA obtained from wild-type and RasGEF1b-cKO BMDMs that were left untreated or treated with LPS for 4 h. After quality trimming, a mean of 94.25% of reads mapped successfully to unique regions of the mouse reference genome. An average read depth above 2.1 × 10^7^ reads/sample was obtained with a range of 2.14 × 10^7^ to 2.78 × 10^7^ reads mapped uniquely (Supplementary Table S2). We used DEseq2 analysis to test for differential gene expression between unstimulated and stimulated cells of each genotype, using a fold-change (FC) 2 and false-discovery rate (FDR) < 0.05 as criteria for significance. LPS treatment led to differential expression of 5017 and 4,914 genes in WT and RasGEF1b-cKO macrophages, respectively (Supplementary Fig. S2). A greater number of genes were up-regulated (2654) than down-regulated (2363) in LPS-stimulated WT macrophages when compared to untreated control cells. Gene ontology (GO) analysis indicated that the top 10 significantly enriched biological processes for nearly all significantly induced genes in WT BMDMs were associated with the inflammatory response (e.g., immune system process, innate immune response, response to lipopolysaccharide), as expected (Supplementary Fig. S3). This confirms the accuracy of our RNA-seq analysis.

**Figure 3:**
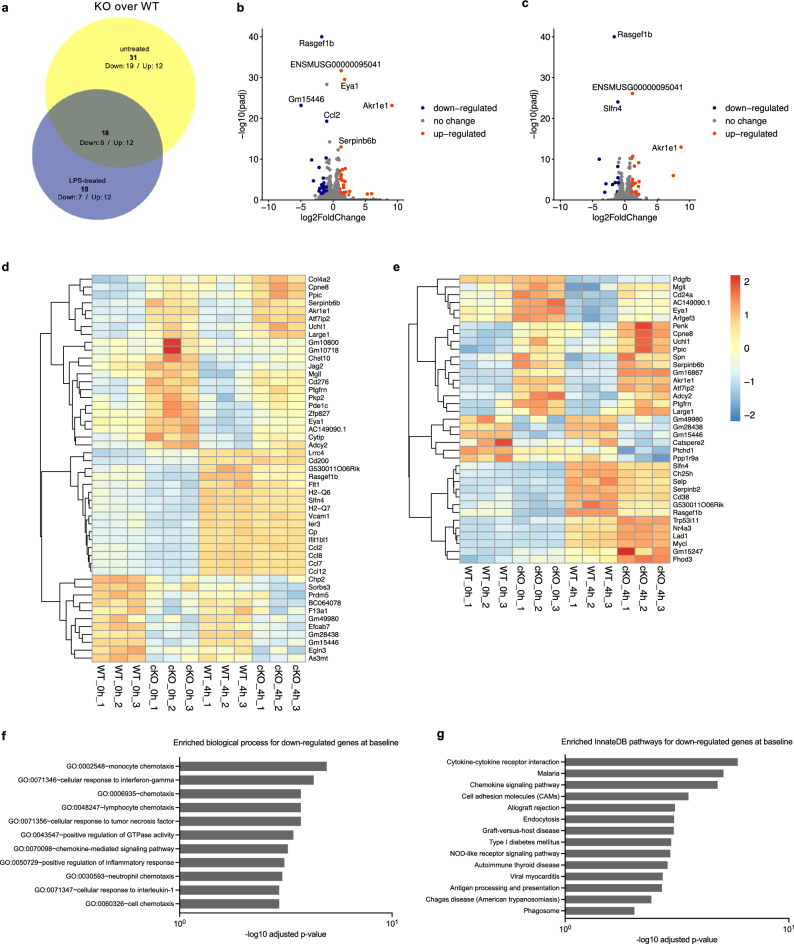
RNA-seq analysis in wild-type and RasGEF1b-cKO macrophages (n = 3). (**a**) Two-circle Venn-diagram showing the number of differentially expressed genes (DEGs) (fold change ≥ 2) in the knock-out BMDMs under basal (untreated) conditions and after LPS stimulation (LPS-treated). DEGs overlapping in both untreated and LPS-treated RasGEF1b-cKO (KO) macrophages are shown in the middle. (**b**, **c**) Volcano plots depicting DEGs in untreated and LPS-treated RasGEF1b-cKO BMDMs. (**d**, **e**) Heatmaps showing the DEGs in untreated (0 h, panel d) and LPS-treated (4 h, panel e) RasGEF1b-cKO macrophages, as compared with wild-type (WT) macrophages, grouped by hierarchical clustering. GO-term (**f)** and pathway analysis (**g**) on genes significantly down-regulated in the knock-out cells. The enriched biological process or pathways identified in RasGEF1b-cKO macrophages are shown and ranked according to the threshold of Benjamini-adjusted *p*-value < 0.01.

Next, we sought to identify which genes in either untreated or LPS-treated conditions were affected by the absence of RasGEF1b. We observed a total of 49 differentially expressed genes (DEGs) at the baseline untreated condition. Twenty-five genes were down-regulated, while 24 were up-regulated in the untreated knock-out macrophages (Fig. 3a,b and Supplementary Table S3). In the LPS-treated condition, we identified a total of 37 DEGs, with 13 genes down-regulated and 24 genes up-regulated (Fig. 3a,c and Supplementary Table S4). Of the identified DEGs, six genes were down-regulated in both untreated and LPS-treated KO cells (*Gm15446*, *Gm28438*, *Slfn4*, *Rasgef1b*, *G530011O06Rik*, *Gm49980*) while 12 genes (*Ppic*, *AC149090.1*, *Large1*, *Serpinb6b*, *Uchl1*, *Adcy2*, *Mgll*, *Ptgfrn*, *Cpne8*, *Eya1*, *Atf7ip2*, *Akr1e1*) were up-regulated (Fig. 3d, e). Thus, the absence of RasGEF1b in macrophages causes a disturbance of the expression of a moderate number of genes under steady-state and LPS-stimulated conditions.

### Functional enrichment analysis of differentially expressed genes

We wanted to obtain insights into potential biological processes implicated in macrophage biology that are affected by the absence of RasGEF1b. To this end, we analyzed the DEG sets for enrichment of Gene Ontology (GO) terms using the high-throughput and integrated data-mining environment DAVID^29^. Using the DEGs as input, including *Rasgef1b*, we found that only the down-regulated genes in non-activated macrophages were enriched for GO terms. Eleven GO categories were significantly enriched with an FDR < 0.01 (Fig. 3f). Interestingly, only eight genes, including *Rasgef1b*, were part of the enriched gene sets. While *Ccl2*, *Ccl7*, *Ccl8,* and *Ccl12* were identified in all of these gene sets, *Flt1* was identified in chemotaxis and monocyte chemotaxis, *H2-Q7* in cellular response to interferon-γ, *Vcam1* in cellular response to tumor necrosis factor, and *Rasgef1b* in positive regulation of GTPase activity. Thus, GO analysis indicated that genes involved in the chemotaxis, cellular response to cytokines, and positive regulation of GTPase activity exhibit altered expression in RasGEF1b-cKO macrophages at the baseline. KEGG pathway enrichment analysis ^32–34^ using the down-regulated genes as input for the InnateDB platform suggested involvement of a variety of immune-related pathways, including infectious (malaria, viral myocarditis, Chagas disease), autoimmune (autoimmune thyroid disease, type I diabetes), and alloimmune (graft-versus-host, allograft rejection) diseases, as well as cytokine and chemokine signaling (Fig. 3g).

### Validation of candidate genes

**Figure 4:**
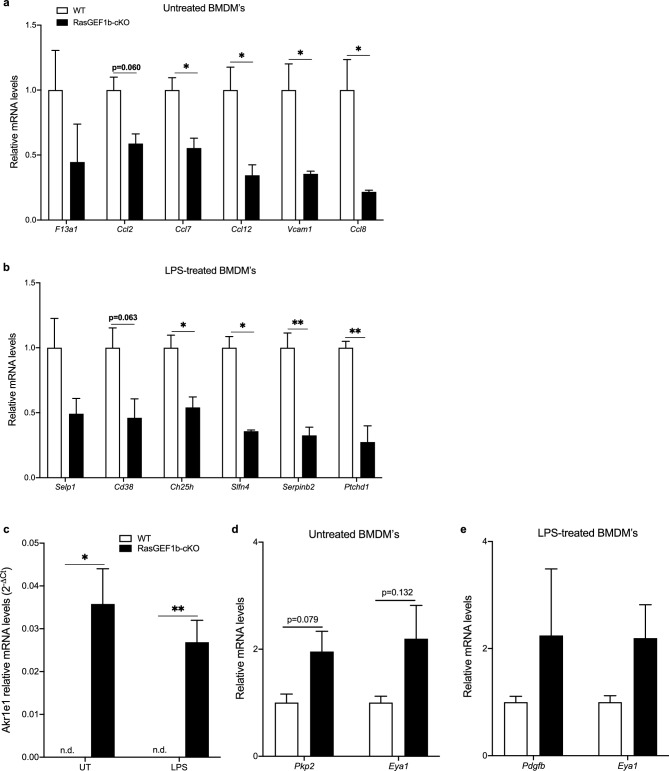
Validation by RT-qPCR of selected differentially expressed genes. Graphs show the relative mRNA expression of the genes differentially down-regulated in RasGEF1b-cKO (KO) macrophages (**a**) under basal conditions. Gene expression levels were normalized to the reference gene *Rpl32* and calibrated to untreated wild-type (WT) cells. (**b**) DEGs in the KO macrophages after treatment with LPS for four hours (n = 3 per genotype). Gene expression levels were normalized to the reference gene *Rpl32* and calibrated to LPS-treated wild-type (WT) cells. Graphs *c–e* show the relative mRNA expression of the genes identified as upregulated by RNA-seq analyses in RasGEF1b-cKO (KO) macrophages*.* Gene expression levels were normalized to the housekeeping gene *Rpl32* and calibrated to untreated (**a,** **d)** or LPS-treated (**b,** **e**) wild-type (WT) cells. Bar represent mean ± SEM. * and ** indicate statistical significance as *p* < 0.05 and *p* < 0.01, respectively. *Ccl2*, *-7*, *-8*, *-12*, C–C Motif Chemokine Ligand; *Cd38*, CD38 antigen; *Ch25h*, Cholesterol 25-Hydroxylase; *F13a1*, Coagulation Factor XIII A Chain; *Ptchd1*, Patched Domain Containing 1; *Serpinb2*, serine protease inhibitor B2; *Selp1*, Selectin platelet; *Slfn4*, Schlafen 4; *Vcam1*, Vascular cell adhesion molecule 1; *Akr1e1*, aldo–keto reductase family 1, member E1; *Eya1*, EYA Transcriptional Coactivator And Phosphatase 1; *Pdgfb*, Platelet Derived Growth Factor Subunit B; *Pkp2*, Plakophilin 2.

We obtained fresh BMDMs from WT and RasGEF1-cKO mice to obtain independent biological RNA samples for the validation of a subset of the DEGs. As GO analyses revealed that only downregulated genes were significantly represented, while no upregulated genes showed significant representation in any biological processes, we selected genes for validation based on the enrichment of GO biological processes among downregulated genes in macrophages. We evaluated the expression of 12 genes that exhibited decreased expression in the knock-out cells in untreated and LPS-treated macrophages. Except for *Ptchd1*, the genes were selected for their previously reported roles in macrophage biology and the inflammatory response ^35–42^. Nine genes (*Ccl7, Ccl8, Ccl12, Vcam1*, *Ch25h*, *Serpinb2*, *Cd38*, *Slfn4*, and *Ptchd1*) were confirmed to be significantly down-regulated, compared to wild-type, while *F13a1*, *Ccl2, and Selp1* exhibited a comparable trend of down-regulation as in the RNA-seq data that did not reach statistical significance (Fig. 4a,b; Supplementary Fig. S5). We also validated the expression levels of four genes that had exhibited up-regulated expression in the knock-out cells by RNA-seq. Remarkably, expression of the gene encoding aldo–keto reductase family 1, member E1, (*Akr1e1*) was completely absent in WT macrophages but strongly and significantly up-regulated in cells deficient for RasGEF1b (Fig. 4c). The expression of *Pkp2*, *Eya1,* and *Pdgfb* expression indicated a trend of up-regulation consistent with the RNA-seq data, but the results showed high variability and did not reach statistical significance in the RT-qPCR analyses (Fig. 4d,e). Therefore, the mRNA levels of DEGs measured by RT-qPCR exhibited a pattern highly similar to that of the RNA-seq data, with some minor statistical differences.

### RasGEF1b mediates the regulation of both basal and signal-dependent gene expression

**Figure 5:**
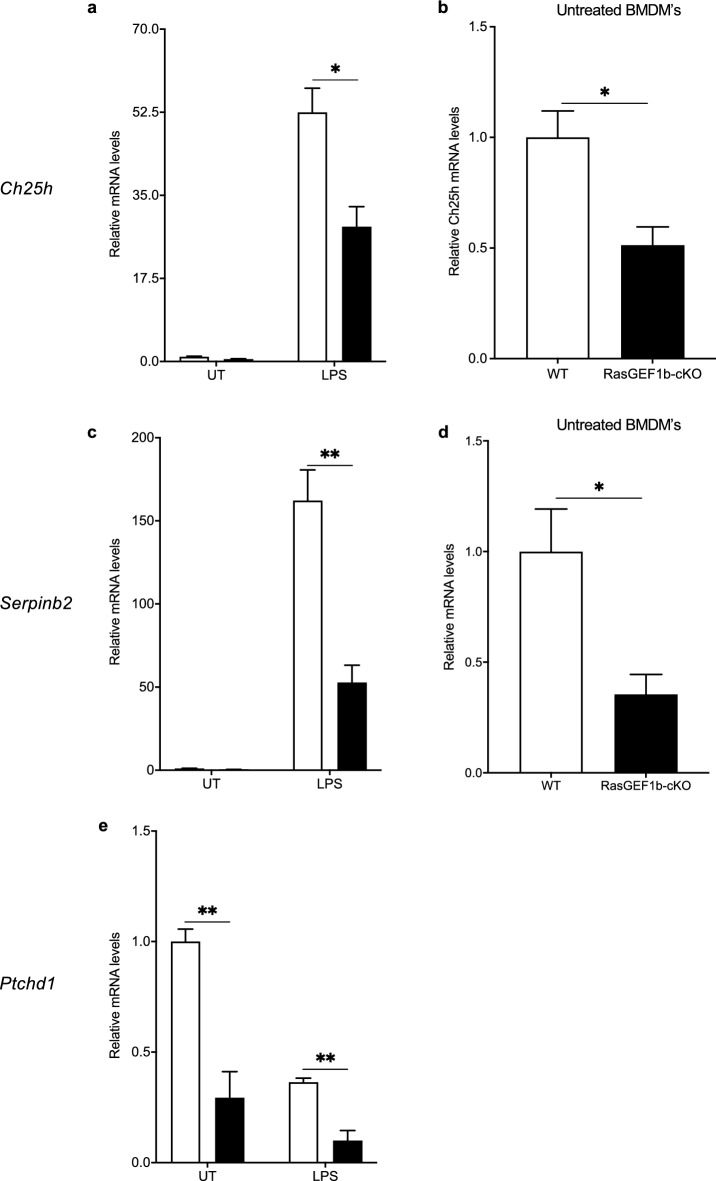
RasGEF1b-cKO macrophages exhibit decreased expression of LPS-responsive genes at baseline. The expression of *Ch25h*, *Serpinb2* and *Ptchd1* in a 2-time point experiment at 0 h (untreated) and 4 h after LPS stimulation (LPS-treated) are shown in *a*, *c* and *e,* respectively. Expression of *Ch25h* and *Serpinb2* are also shown for RasGEF1b-cKO (KO) and wild-type (WT) macrophages under untreated conditions in *b* and *d*, respectively. Data are mean ± SEM (n = 3 per condition). Unpaired t-tests for multiple comparisons between KO and WT macrophages: *p < 0.05, **p < 0.01. *Ch25h*, Cholesterol 25-Hydroxylase; *Serpinb2*, serine protease inhibitor B2; *Ptchd1*, Patched Domain Containing 1*.* White columns show WT samples, black columns show KO samples.

While validating the RNA-seq DEGs whose expression was significantly affected by the absence of RasGEF1b in the LPS-treated cells, we noticed that their expression was also significantly decreased in the untreated macrophages as revealed by the RT-qPCR analyses (Fig. 5a–e). These genes were *Ch25h*, *Serpinb2*, and *Ptchd1*. This observation indicates that the same effect of the absence of RasGEF1b on gene expression observed in unstimulated cells (baseline gene expression) is also seen in the macrophages after treatment with LPS, indicating that RasGEF1b may be required to drive and sustain the steady-state expression of genes that are regulated during inflammatory stimulation.

### Ectopic expression of RasGEF1b results in the transcriptional activation of Serpinb2

**Figure 6:**
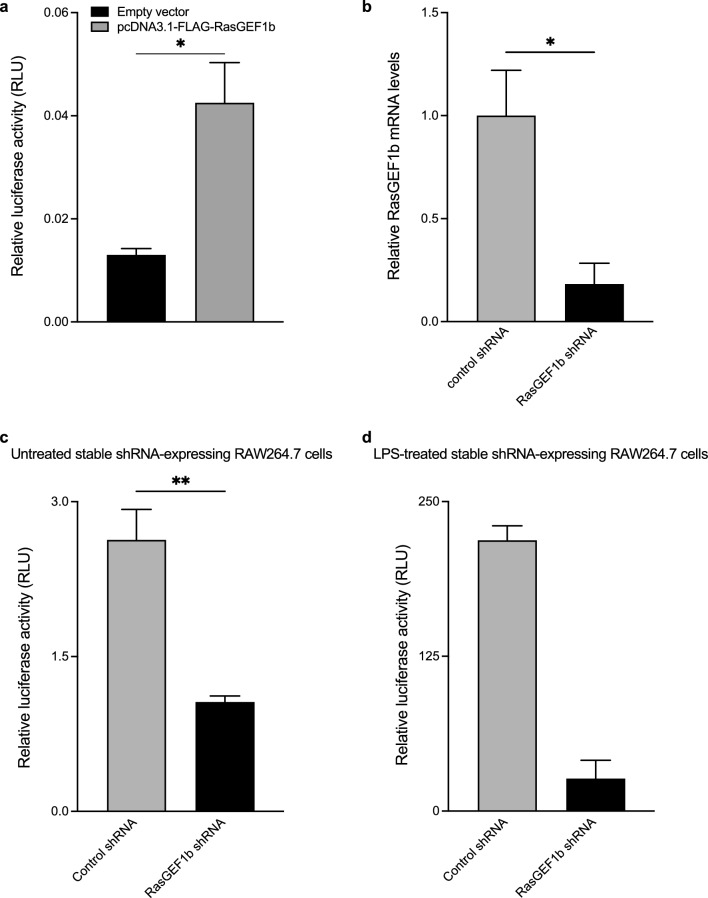
Effects of RasGEF1b overexpression or knock-down on SerpinB2 promoter transcriptional activation. (**a**) RasGEF1b increases the activity of the SerpinB2 promoter. HEK293 cells were transfected with an empty vector or an expression construct encoding FLAG-RasGEF1b (NM_145839), along with pGLmP-539 SerpinB2 promoter-luciferase reporter and pRL-TK reporter plasmids. Cell lysates were collected after 24 h and assayed for luciferase activity. Transfection experiments were performed ≥ 4 times in independent biological experiments. Data represent the mean ± SEM. Statistical significance was determined for RasGEF1b encoding plasmid compared to pFLAG-CMV4 vector by Student t-test (*p < 0.05). (**b**) RT-qPCR analysis showing knock-down of RasGEF1b mRNA in shRNA-expressing RAW264.7 cells. Gene expression levels were normalized to the reference gene *Rpl32* and calibrated to control shRNA-expressing cells (n = 3 independent experiments). (**c**, **d**) Luciferase assays showing SerpinB2 transcriptional activation in puromycin-resistant shRNA control or shRNA RasGEF1b-expressing RAW264.7 cells. Transfections were performed in 24-well plates containing 400 ng and 100 ng of pGLmP-539 and pRL-TK per well, respectively. Twenty-four hours post-transfection, cells were left untreated or treated with LPS 100 ng/ml. Cell lysates were collected after 18 h and assayed for luciferase activity (n = three and two independent experiments for *c* and *d*, respectively). Bars represent mean ± SEM. * and ** indicate statistical significance as *p* < 0.05 and *p* < 0.01, respectively.

It has been shown that constitutive Serpinb2 mRNA expression in macrophages is tightly regulated by its proximal promoter^22^. Thus, we investigated whether RasGEF1b induced Serpinb2 transcription through its promoter by using luciferase assays with a SerpinB2 reporter construct pGLmP-539. We found that RasGEF1b activated the *Serpinb2* 5′ regulatory region by 3.26 fold above basal luciferase activity (Fig. 6a).

### Knockdown of RasGEF1b in RAW264.7 macrophages inhibits both the constitutive and LPS-induced transcriptional activation of Serpinb2

To complement the overexpression-based experiments, we utilized lentiviral vectors encoding control shRNA and RasGEF1b shRNA to knock down the murine RasGEF1b mRNA transcript, allowing us to further assess the effects on SerpinB2 transcriptional activation. Using RAW264.7 cells transduced with control or RasGEF1b shRNA lentiviruses, we confirmed the knockdown of RasGEF1b by 81.77% through RT-qPCR experiments (Fig. 6b). Notably, the knockdown of RasGEF1b in macrophages resulted in decreased luciferase activity of the *Serpinb2* promoter gene reporter under both unstimulated and LPS-stimulated conditions (Fig. 6c, d), providing further evidence of the role of RasGEF1b in gene expression under basal and inflammatory conditions in macrophages.

## Discussion

In the present work, we have provided genetic evidence revealing a role for RasGEF1b in the regulation of gene expression in macrophages. Using high-throughput transcriptome sequencing (RNA-seq), we demonstrate that the absence of RasGEF1b disturbs the expression of genes under unstimulated conditions and after lipopolysaccharide stimulation of primary macrophages.

The transcriptomics analyses identified a modest number of differentially expressed genes, i.e., a total of 68 in unstimulated and LPS-treated RasGEF1b-cKO macrophages. Regarding the timepoint chosen for LPS treatment, it is important to mention that four-hour time point is commonly used in immune response studies because it captures the early and critical phase of macrophage response to LPS. Many immune-related genes, including pro-inflammatory cytokines, chemokines, and signaling molecules, are upregulated during this time frame. This choice balances the need to capture significant gene expression changes with practicality, as using a common 4 h time point allows for easier comparisons between different studies while minimizing experiment duration.

Whereas the DEGs identified by the RNA-seq approach as down-regulated in LPS-treated macrophages or up-regulated in both conditions did not exhibit an enrichment for Gene Ontology (GO) terms, the genes down-regulated in unstimulated KO macrophages were enriched for biological processes implicating a role in cell chemotaxis, cellular response to cytokines, and positive regulation of GTPases. Specifically, the genes encoding the chemokines Ccl2, Ccl7, Ccl8, and Ccl12 were identified in all categories that included chemotaxis, cell chemotaxis, and monocyte, lymphocyte and neutrophil chemotaxis; cellular responses to IFN-γ, TNF, and IL-1; positive regulation of GTPase activity and inflammatory response; and chemokine-mediated signaling pathway. VCAM-1, Flt-1, and H2-Q7 were identified respectively in cellular response to TNF, chemotaxis and monocyte chemotaxis, and cellular response to IFN-γ. Despite these being all genes commonly regulated during inflammation, it is interesting to note their differential expression at baseline in the knock-out cells. Nonetheless, these analyses are highly suggestive of the involvement of RasGEF1b in macrophage functions by regulating the constitutive expression of these genes.

The reduced expression of *Ccl2*, *Ccl7*, *Ccl8,* and *Ccl12* was confirmed by RT-qPCR in the unstimulated RasGEF1b-cKO macrophages. These chemokines belong to a cluster of CC chemokines encoding genes in the mouse chromosome 11, and are referred to as monocyte chemoattractants as they are implicated in the recruitment of monocytes to sites of trauma or infection. Abrogation of Ccl2 (MCP-1, Monocyte Chemoattractant Protein-1) has been shown to cause a dramatic decrease in the migration of monocytes into the peritoneal cavity of mice challenged intraperitoneally with thioglycollate^35^. Ccl7 (MCP-3) promotes the recruitment of innate immune cell types, such as monocytes and neutrophils, to sites of infection by bacteria or viruses^36^. Accordingly, mice devoid of Ccl7 present decreased inflammation and poor pathogen control^37^. Murine Ccl8 (MCP-2) is a chemotactic protein that, unlike most CC chemokines including Ccl2, Ccl7 and Ccl12, does not signal through Ccr2 but instead acts as an agonist for Ccr8^38^. Ccl12 (MCP-5) is structurally most closely related to human CCL2/MCP-1 with 66% identity^43^. Despite being expressed constitutively in the thymus and lymph nodes, it can be induced under inflammatory conditions in activated macrophages^44^. Ccl12 acts chemotactically to attract macrophages, eosinophils, and lymphocytes but not neutrophils^45^.

Expression of *Vcam1* encoding the Vascular Cell Adhesion Molecule 1 (VCAM-1) was down-regulated 3.3-fold in the unstimulated RasGEF1b-deficient macrophages as identified in the RNA-seq analyses and further confirmed by RT-qPCR validation experiments, which showed a 2.8-fold reduction in the knock-out cells. The gene is a member of the Immunoglobulin (Ig) superfamily and encodes a cell-surface protein that is inducible and preferentially expressed in a cytokine-dependent manner by endothelial cells^39,46,47^. Expression of VCAM-1 has been demonstrated in other cell types and different organs of mice^48^, and it has been shown that the protein is constitutively expressed in myeloid cells confined to hematopoietic tissues or by proliferating myeloid cells in culture^49^. Interestingly, macrophages in the splenic red pulp express VCAM-1 and operate to retain hematopoietic stem and progenitor cell in the spleen^50^. These are important observations as the molecular mechanism by which VCAM-1 expression is regulated in VCAM-1-positive macrophages remains to be established.

The hierarchical clustering of the genes differentially expressed indicated a particular group of six down-regulated LPS-responsive genes. These genes were *Ch25h*, *Serpinb2*, *Slfn4*, *Selp*, *Cd38*, and *G530011O06Rik*. Except for *G530011O06Rik*, we carried out RT-qPCR analyses for validation and could confirm the differential expression of the three transcripts at the top of the list. *Ch25h* encodes the cholesterol 25-hydroxylase, an enzyme that catalyzes the synthesis of 25-hydroxycholesterol from cholesterol and molecular oxygen. Ch25h expression is moderate in non-activated immune cells, but it is highly induced by inflammatory stimuli such as Toll-like receptor ligands and exerts a role in the innate immune response^40,51^. *Serpinb2* encodes a serine protease implicated in the degradation of the extracellular matrix through the activation of plasminogen^52^. Its expression is highly induced by LPS in macrophages^41,53^. It can also be induced by other inflammatory mediators such as cytokines^52^, and its expression seems to be regulated in a cell-type-specific manner^54^. *Slfn4* belongs to the *Schlafen* family of genes, comprised of ten and six members in mice and humans, respectively^55^, which are mostly expressed in cells of the immune system. The expression of *Schlafen* genes can also be altered during developmental processes. This is the case for *Slfn4*, which is down-regulated during macrophage differentiation^42^. The expression of *Slfn4* is strongly induced by LPS and poly-(I:C), a TLR3 agonist and by interferons (IFNs) in macrophages. Correspondingly, the putative promoter region harbors IFN-responsive elements^42^.

Our studies further demonstrate that RasGEF1b can regulate the baseline expression of genes whose LPS-induced or -down-regulated expression was impaired in the RasGEF1b knock-out macrophages. Deficiency of RasGEF1b in macrophages resulted in significantly diminished mRNA levels of *Ch25h*, *Serpinb2*, and *Ptchd1* in the absence of LPS stimulation. Our luciferase assays with a SerpinB2 reporter construct showed that RasGEF1b ectopic expression resulted in significant transcriptional activation of the gene, suggesting that RasGEF1b positively regulates *Serpinb2* expression through its promoter. When we employed knockdown experiments using lentiviral vectors, our results demonstrated that knockdown of RasGEF1b in RAW264.7 macrophages significantly decreased the luciferase activity of the promoter gene reporter, both in unstimulated and LPS-stimulated conditions. This indicates that RasGEF1b acts as a key facilitator in the constitutive and LPS-induced transcriptional activation of *Serpinb2*. The transcriptional regulation of SerpinB2 is of particular interest, as previous studies have shown that its mRNA expression in macrophages is tightly controlled by its proximal promoter, with direct binding of C/EBP- β to this region. Deficiency of C/EBP-β abrogates both constitutive and LPS-induced SerpinB2 expression^22^. Our findings provide novel insights into the role of RasGEF1b in the transcriptional activation of *Serpinb2* and suggest that RasGEF1b plays an important role in maintaining the constitutive and LPS-induced expression of the gene in macrophages. Although the mechanisms that fully regulate SerpinB2 expression remain elusive in the literature, and there is a dearth of information regarding the involvement of a RasGEF in the regulation of SerpinB2 gene expression, we can only speculate about a mechanistic link between RasGEF1b and SerpinB2 expression. In one study, it was demonstrated that Ras GTPase induces phosphorylation of C/EBP-beta, and the activated C/EBP-beta interacts with the transcriptional mediator complex, providing evidence that Ras signaling plays a role in the final step of differential gene expression of C/EBP-beta target genes^56^. Therefore, one might speculate that Ras activation by RasGEF1b in cells could lead to C/EBP-beta activation, thereby regulating gene transcription. Further investigation of downstream signaling events related to RasGEF1b in macrophages is warranted to explore this possibility.

The decreased gene expression observed in RasGEF1b-deficient macrophages under resting conditions may have implications for homeostatic signaling, which in turn could affect the macrophage response to danger signals. This suggests that RasGEF1b-mediated regulation of gene expression in macrophages may play a role in maintaining macrophage function and responsiveness during both basal and inflammatory conditions. For example, Ch25h is highly expressed in peritoneal macrophages, and besides being induced by TLR ligands, it is also an interferon-stimulated gene (ISG), and its primary product 25-hydroxycholesterol (25HC) exerts antiviral activity^57^. Collectively, our findings suggest that RasGEF1b mediates the regulation of both basal and signal-dependent gene expression in macrophages, and its absence leads to decreased expression of target genes under both basal and inflammatory conditions.

The up-regulation of the genes identified by RNA-seq in the macrophages devoid of RasGEF1b is particularly puzzling, especially because these genes were not enriched for any biological process or pathway. The RT-qPCR validation for most of these genes showed high variability and did not reach statistical significance. We examined the expression of *Akr1e1*, *Pkp2*, and *Eya1*, genes that are typically expressed at low or negligible levels in macrophages. Confirming their expression levels could shed light on the mechanisms governing their regulation and offer insights into the functional role of RasGEF1b in gene regulation within macrophages. As for *Pdgfb*, this gene is induced by LPS in macrophages, and it plays a critical role in chemotaxis and growth stimulation. However, of particular note is the gene *Akr1e1* encoding the aldo-ketoreductase member e1, with a striking 400- to 500-fold increase in macrophages lacking RasGEF1b. The validation by RT-qPCR showed intermediate cycle threshold (Cq) values (< 24.5) in the knock-out cells (average Cq values 23.28 and 23.32 in the untreated and LPS-treated cells, respectively), and Cq higher than 40 in cDNA samples of wild-type macrophages. This indicates that *Akr1e1* is tightly repressed under basal conditions and prevented from being expressed when RasGEF1b is present. Akr1e1 catalyzes the NADPH-dependent reduction of 1,5-anhydro-D-fructose (AF), a metabolite of the alternative glycogen degradation pathway, to 1,5-anhydro-D-glucitol (AG)^58^. We are currently investigating the molecular mechanisms by which *Akr1e1* is derepressed in the absence of RasGEF1b in macrophages. It will also be interesting to perform studies to evaluate the aldoketoreductase activity in RasGEF1b-cKO macrophages and the outcomes of the likely concomitant increase in the concentration of AG under inflammatory conditions induced by LPS^59^.

Studies have demonstrated that RasGEF1b can activate Rap2A through its GEF activity by facilitating the conversion of Rap2A from an inactive GDP-bound state to an active GTP-bound state^11^. This activation of Rap2A by RasGEF1b can initiate downstream signaling cascades that play a role in regulating various cellular processes. Additionally, in macrophages, the expression levels of Rap2A seem to be tightly regulated to maintain balanced immune responses, involving the transcription factor NF-κB^12^. However, the potential cooperative relationship between RasGEF1b and Rap2A in gene expression and cellular signaling requires further investigation for a comprehensive understanding.

In conclusion, our study provides genetic evidence that macrophages lacking RasGEF1b exhibit altered expression of several genes under basal and stimulated conditions with functions in cell chemotaxis and response to cytokines. Further studies will determine the mechanisms underlying the precise function of RasGEF1b in individual gene expression. Exploring the molecular mechanisms underlying the regulation of gene expression by RasGEF1b may provide valuable insights into the complex interplay between signaling pathways and gene expression in macrophages and other cell types. It will also be important to use the knock-out mouse generated here to investigate the expression of the DEGs and to explore whether loss of RasGEF1b-mediated positive regulation of LPS-responsive genes impacts the inflammatory response in vivo.

### Supplementary Information

Supplementary Information.

## Data Availability

The RNA-seq datasets are available in the National Center for Biotechnology Information Gene Expression Omnibus repository, accession number GSE190801. The hyperlink to the datasets is given as follows: https://www.ncbi.nlm.nih.gov/geo/query/acc.cgi?acc=GSE190801
