# Supplementary material for: Transcriptomic analysis reveals that RasGEF1b deletion alters basal and LPS-induced expression of genes involved in chemotaxis and cytokine responses in macrophages
Source: Sci Rep. 2023 Nov 10;13:19614. doi: 10.1038/s41598-023-47040-9 (PMC10638313; doi:10.1038/s41598-023-47040-9)
Supplement: Supplementary file 1 — Supplementary Information. [file 41598_2023_47040_MOESM1_ESM.pdf]

Supplementary information.

Transcriptomic analysis reveals that RasGEF1b deletion alters basal and LPS-induced expression of genes involved in chemotaxis and cytokine responses in macrophages

Heliana B. Fernandes, Isadora Mafra de Oliveira, Thomas S. Postler, Sérgio Q. Lima, Cícera A. C. Santos, Michaelle S. Oliveira, Felipe B. Leão, Sankar Ghosh, Maria C. Souza, Warrison Andrade, Aristóbolo M. Silva

Supplementary Fig.S1

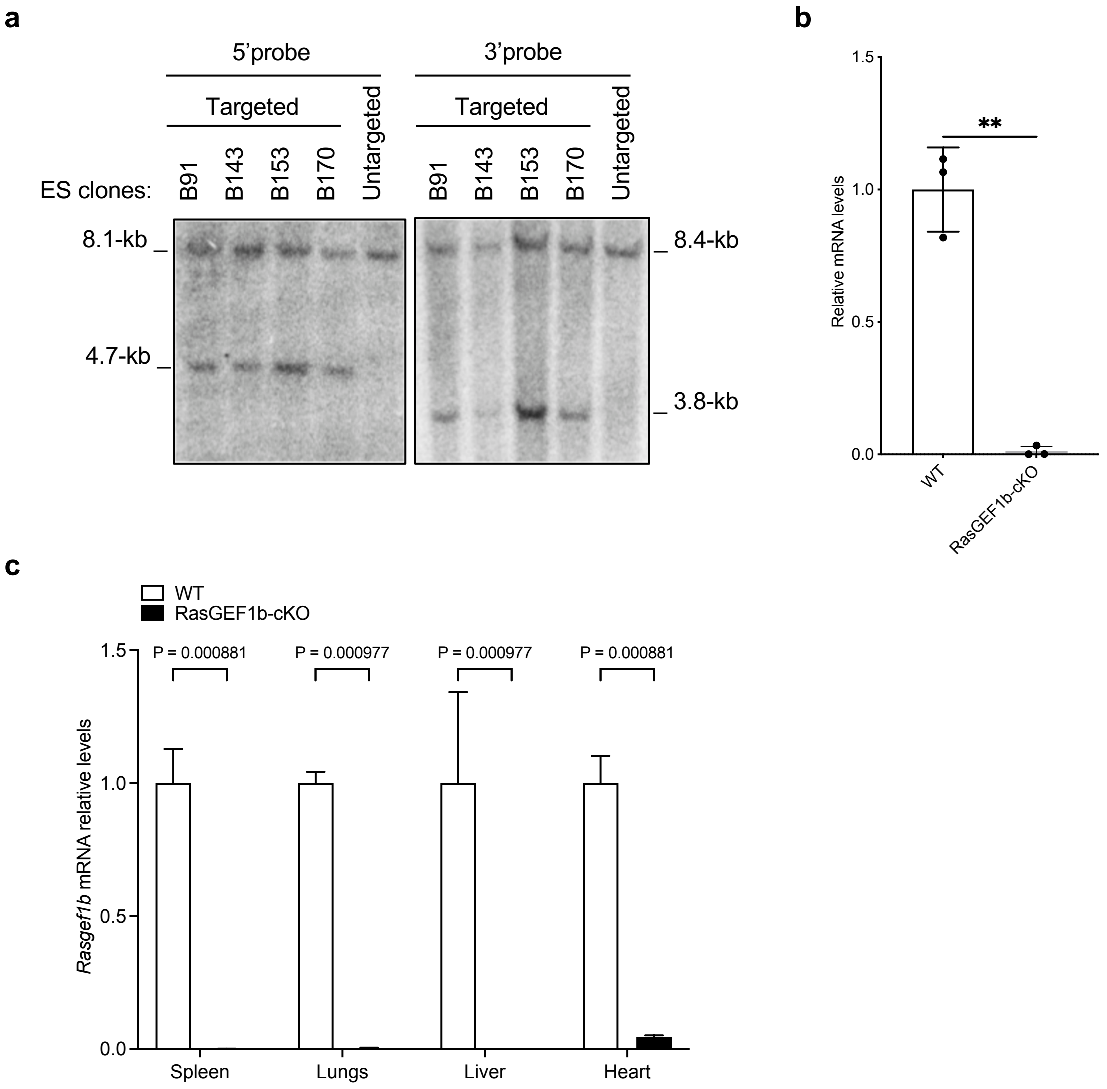

Supplementary Fig.S1: (a) Representative southern blot analyses with the 5’ and 3’ probes indicated in panel A of figure 2 showing the correct identification of targeted *Rasgef1b*$^{flox/Frt/Neo}$ allele in ES cells. Genomic DNA from four targeted clones and untargeted ES cells were digested with *Afl*II or *Eco*RI and blotted with probe external to the 5’-targeting arm (left autoradiogram) or 3’-targeting arm (right autoradiogram). RT-qPCR analysis of RasGEF1b in fresh bone marrow stem cells (b), and spleen, lungs, liver, and heart (c) taken from wild-type (WT), and *Rasgef1b*$^{fl/fl}$;CMV-cre mice (RasGEF1b-cKO). Data are represented as mean ± SEM (n = 3-6 per group). ** $p < 0.01$ and p values, Student’s unpaired t-test, relative to the wild type cells are indicated in panels b and c, respectively.

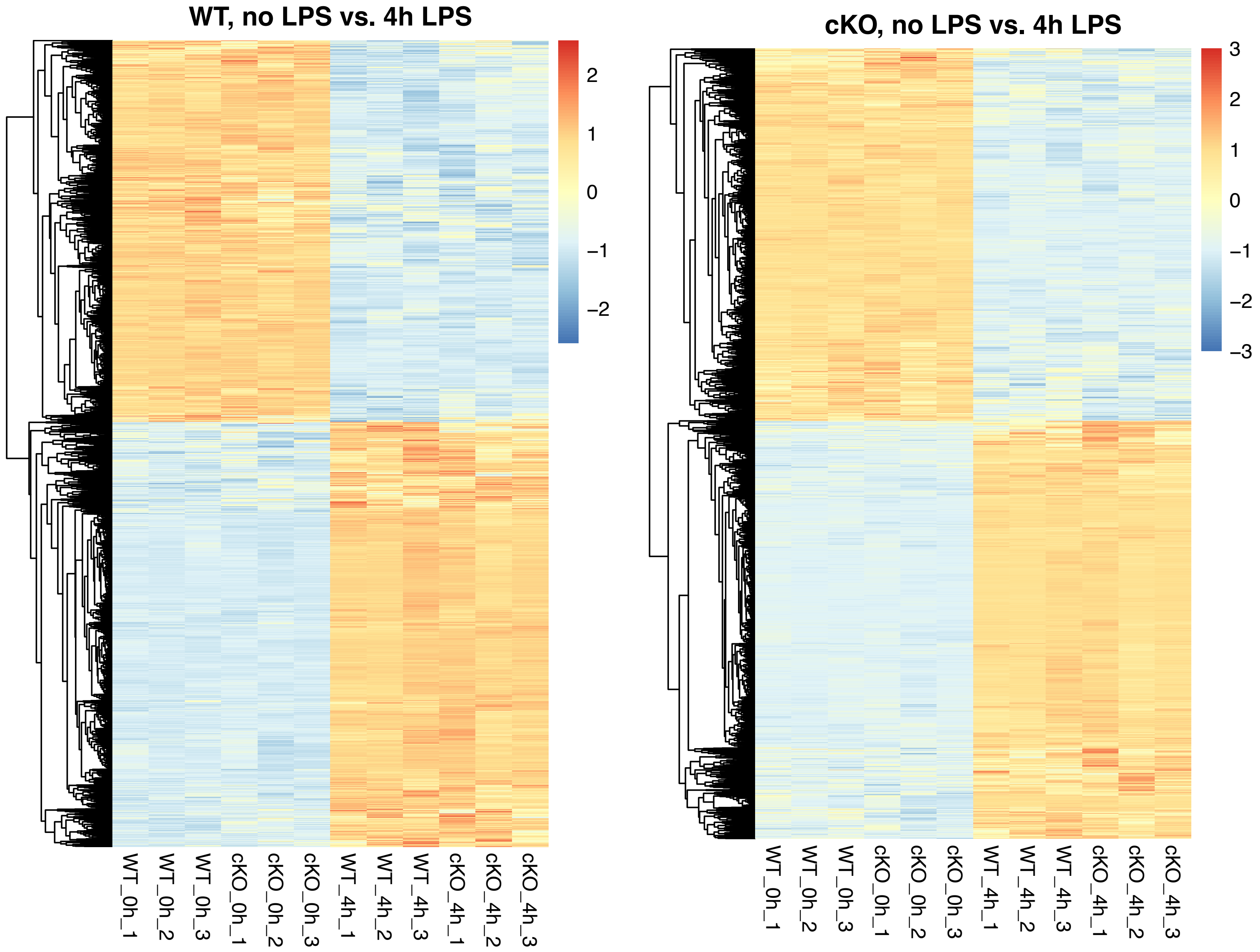

Supplementary Fig.S2: Heat map of transcript profiles from untreated or LPS-treated WT and RasGEF1b-cKO BMDM's. Genes were selected by fold change (Log_2 FC > 1 and < -1 between untreated versus LPS-treated) and adj. P-value (adjusted P-value < 0.05) filtering. Hierarchical clustering and normalized gene counts are shown.

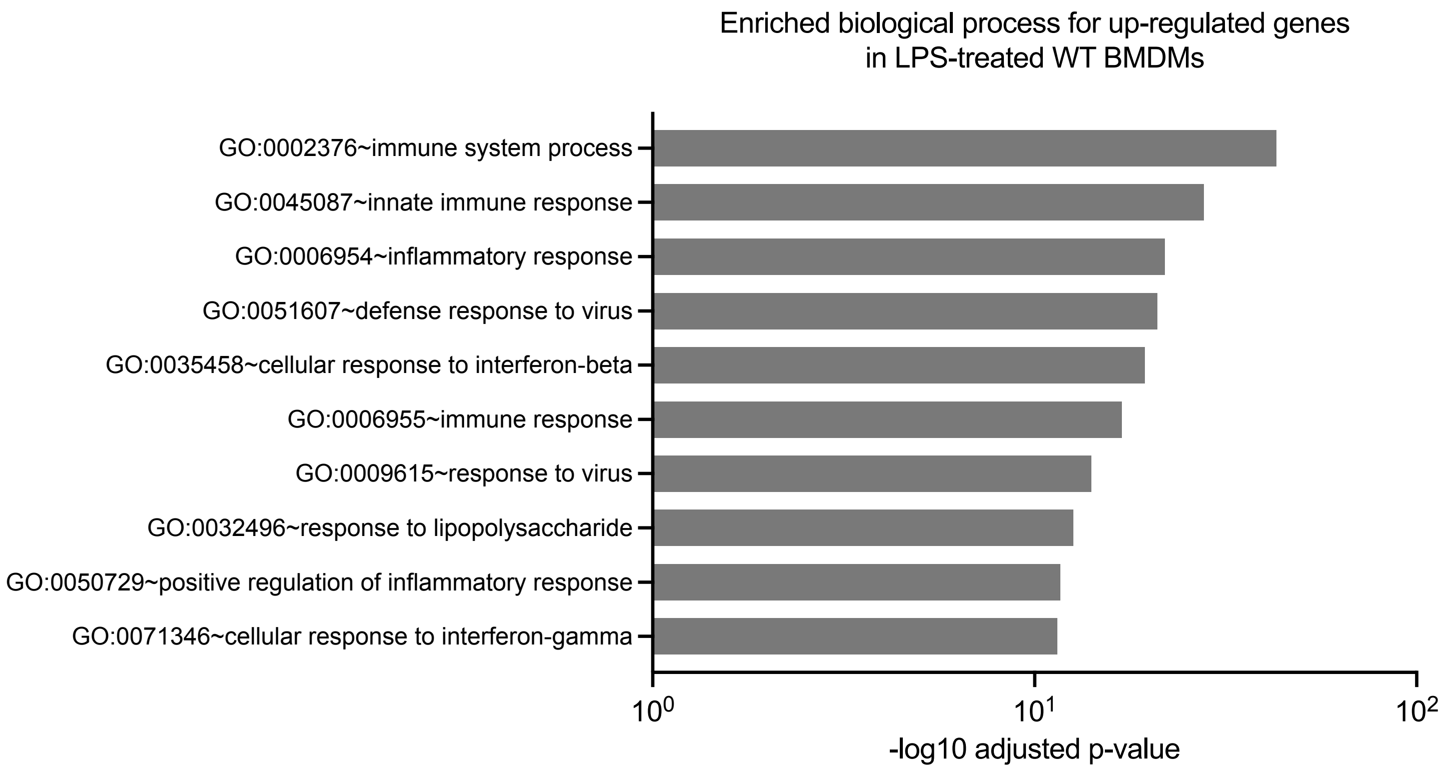

Supplementary Fig.S3: GO analysis for significantly upregulated genes in LPS-stimulated BMDMs showing biological process.

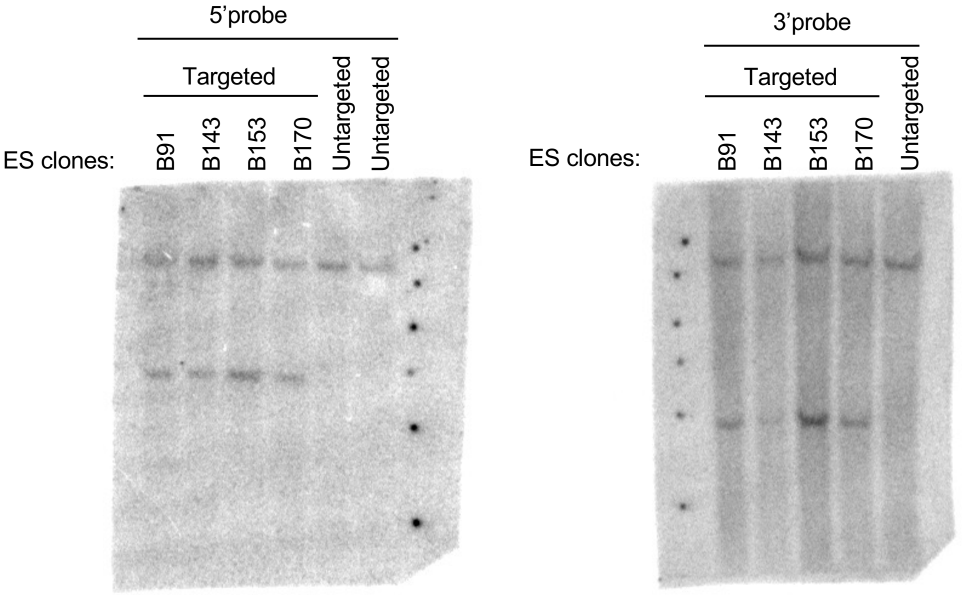

Supplementary Fig.S4: : Scans of the original Southern-blot membranes related to Supplementary Fig.S1a. The dots on the right or left margins in the membranes indicate positions of DNA markers, which are 10, 8, 6, 5, 4, 3kb from top to bottom.

Supplementary Fig.S5

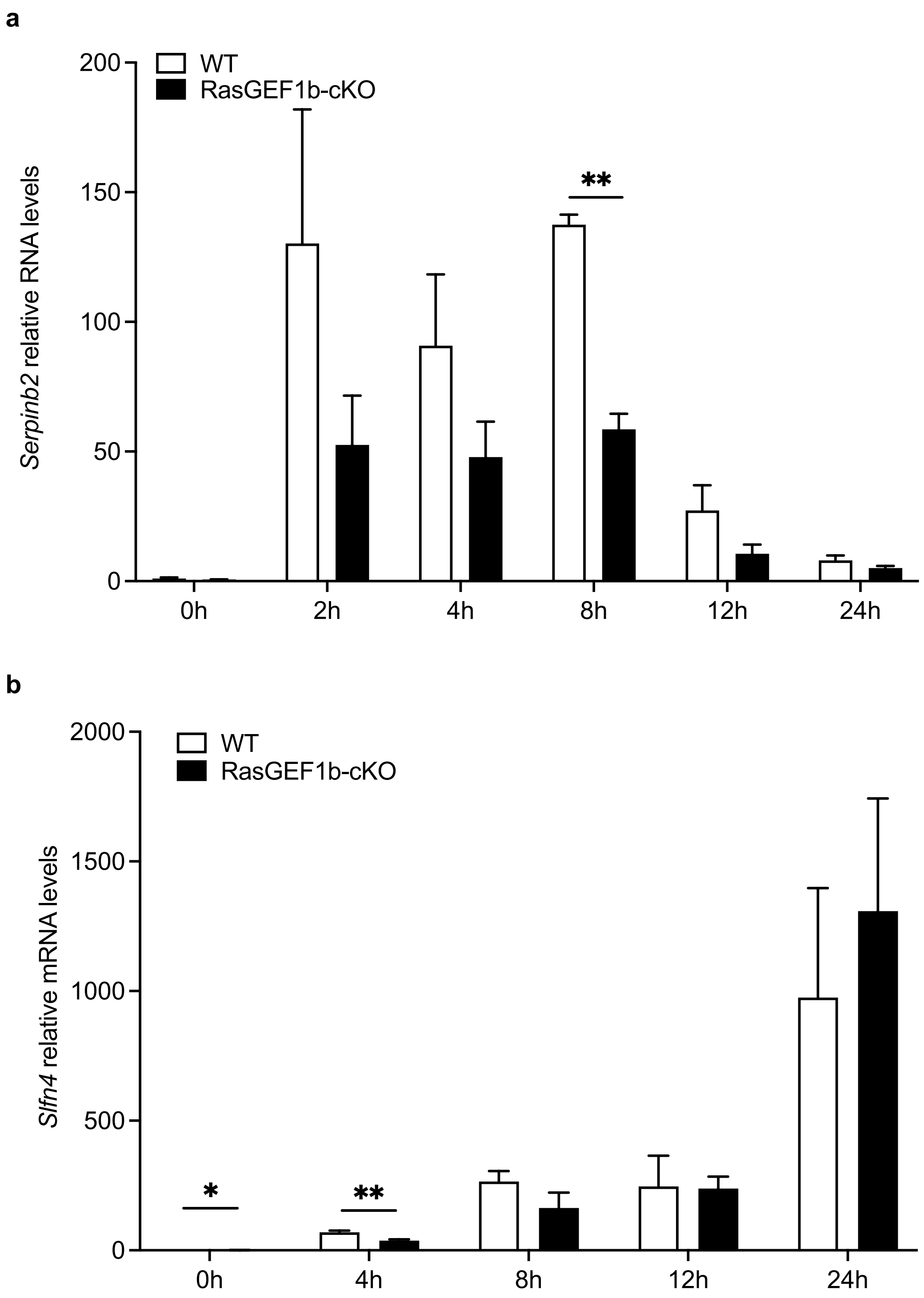

Supplementary Fig.S5: BMDMs from wild-type (WT) and RasGEF1b-cKO mice were treated with LPS 100ng/mL for the time intervals indicated in the figure. The mRNA levels of *SerpinB2* (a) and *Slfn4* (b) were determined. Gene expression levels were normalized to the reference gene *Rpl32* and calibrated to untreated wild-type (WT) cells. Error bars represent the standard deviation from independent cultures from three mice per genotype. A p value (students t-test) relative to the wild type cells of less than 0.05 is indicated by * and less than 0.01 by **.

Table S1: Gene expression of RasGEF1 domain family members in macrophage lineages from BioGPS database (www.biogps.org). Numbers represent gene expression values ± standard deviation.

**Macrophages lineage**	***Rasgef1b***	***Rasgef1a***	***Rasgef1c***
Macrophage bone marrow	1,081.51 ± 103.43	9.05 ± 5.37	4.64 ± 0.00
Microglia	705.93 ± 267.95	7.64 ± 3.86	4.64 ± 0.00
Macrophage peritoneal	428.30 ± 49.25	20.86 ± 8.56	6.77 ± 3.02
Osteoclasts	375.96 ± 20.84	5.89 ± 0.51	4.64 ± 0.00
RAW264.7	283.96 ± 20.45	4.89 ± 0.046	4.64 ± 0.00

Table S2: Quality control and mapping of RNA-seq experiment. Number of reads and percentage (per sample), and read mapping is indicated.

**Sample**	***Rasgef1b* genotype**	**Experimental condition**	**Uniquely mapped reads number**	**Uniquely mapped reads %**
MD01-RNA-01	+/+	untreated	26,984,616	94.95
MD01-RNA-02	+/+	untreated	27,874,638	94.99
MD01-RNA-03	+/+	untreated	23,211,873	94.97
MD01-RNA-04	+/+	LPS-treated	21,437,044	94.49
MD01-RNA-05	+/+	LPS-treated	21,951,395	94.41
MD01-RNA-06	+/+	LPS-treated	22,531,814	94.54
MD01-RNA-07	-/-	untreated	25,924,714	94.80
MD01-RNA-08	-/-	untreated	23,913,677	90.31
MD01-RNA-09	-/-	untreated	25,646,154	94.77
MD01-RNA-10	-/-	LPS-treated	25,413,821	94.26
MD01-RNA-11	-/-	LPS-treated	22,057,497	94.29
MD01-RNA-12	-/-	LPS-treated	21,962,769	94.27

Table S3. Differentially expressed genes under basal conditions.
Shown are genes with FC log2 ≥1 ≤-1 padj < 0.05 in untreated KO/WT BMDMs.

Geneid	baseMean	log2FoldChange	padj	external_gene_name
ENSMUSG00000090015	71.36	-4.98	6.92E-24	*Gm15446*
ENSMUSG00000027962	62.99	-3.33	1.56E-10	*Vcam1*
ENSMUSG00000101939	20.89	-3.05	2.00E-05	*Gm28438*
ENSMUSG00000030865	21.05	-2.18	6.38E-03	*Chp2*
ENSMUSG00000009185	87.49	-2.18	1.11E-08	*Ccl8*
ENSMUSG00000000204	53.14	-2.07	1.52E-03	*Slfn4*
ENSMUSG00000039109	875.31	-1.84	2.71E-02	*F13a1*
ENSMUSG00000087150	20.56	-1.83	2.08E-02	*BC064078*
ENSMUSG00000029913	15.97	-1.82	3.71E-02	*Prdm5*
ENSMUSG00000089809	1819.54	-1.77	7.17E-74	*Rasgef1b*
ENSMUSG00000003617	48.17	-1.77	1.87E-04	*Cp*
ENSMUSG00000072844	62.68	-1.72	3.46E-04	*G530011O06Rik*
ENSMUSG00000060550	43.20	-1.65	1.31E-03	*H2-Q7*
ENSMUSG00000073409	44.70	-1.53	1.57E-02	*H2-Q6*
ENSMUSG00000035352	92.72	-1.53	4.46E-06	*Ccl12*
ENSMUSG00000035105	52.62	-1.50	4.66E-04	*Egln3*
ENSMUSG00000073791	44.49	-1.48	4.41E-04	*Efcab7*
ENSMUSG00000117465	101.00	-1.47	8.46E-03	*Gm49980*
ENSMUSG00000079339	77.38	-1.44	6.44E-05	*Ifit1bl1*
ENSMUSG00000029648	85.39	-1.25	2.96E-03	*Flt1*
ENSMUSG00000035373	2466.08	-1.08	5.05E-11	*Ccl7*
ENSMUSG00000003559	95.20	-1.04	7.43E-04	*As3mt*
ENSMUSG00000022091	115.51	-1.01	2.75E-02	*Sorbs3*
ENSMUSG00000003541	223.08	-1.01	6.06E-03	*Ier3*
ENSMUSG00000035385	5262.25	-1.00	5.14E-20	*Ccl2*
ENSMUSG00000035914	197.52	1.03	8.41E-08	*Cd276*
ENSMUSG00000049939	76.77	1.04	1.83E-03	*Lrrc4*
ENSMUSG00000033174	65.65	1.10	1.07E-02	*Mgll*
ENSMUSG00000004383	494.74	1.15	7.95E-06	*Large1*
ENSMUSG00000026832	606.77	1.18	1.47E-06	*Cytip*
ENSMUSG00000022661	33.35	1.21	2.76E-02	*Cd200*
ENSMUSG00000042842	628.49	1.22	1.00E-13	*Serpinb6b*
ENSMUSG00000095041	4998.22	1.25	2.16E-32	*AC149090.1*
ENSMUSG00000004347	280.64	1.26	6.34E-07	*Pde1c*
ENSMUSG00000024538	145.98	1.26	6.18E-06	*Ppic*
ENSMUSG00000002799	152.11	1.40	1.66E-05	*Jag2*
ENSMUSG00000029223	287.85	1.43	3.52E-08	*Uchl1*
ENSMUSG00000039200	43.15	1.59	2.16E-04	*Atf7ip2*
ENSMUSG00000071064	24.34	1.62	1.62E-02	*Zfp827*
ENSMUSG00000027864	67.31	1.63	8.86E-04	*Ptgfrn*
ENSMUSG00000026080	24.95	1.67	2.99E-02	*Chst10*
ENSMUSG00000025932	779.09	1.78	2.82E-30	*Eya1*
ENSMUSG00000041957	50.50	1.83	3.03E-05	*Pkp2*
ENSMUSG00000021536	98.18	1.90	2.04E-08	*Adcy2*
ENSMUSG00000052560	18.24	1.97	1.15E-02	*Cpne8*
ENSMUSG00000031503	17.28	2.56	8.43E-03	*Col4a2*
ENSMUSG00000075014	2155.29	5.29	3.47E-02	*Gm10800*
ENSMUSG00000095186	89.18	5.98	3.04E-02	*Gm10718*
ENSMUSG00000045410	267.63	9.11	6.92E-24	*Akr1e1*

Table S4. Differentially expressed genes after LPS-stimulation.
Shown are genes with FC log2 ≥1 ≤-1 padj < 0.05 in LPS-treated KO/WT BMDMs.

Geneid	baseMean	log2FoldChange	padj	external_gene_name
ENSMUSG00000090015	32.12	-3.97	1.05E-10	*Gm15446*
ENSMUSG00000091476	15.24	-3.12	1.33E-02	*Catspere2*
ENSMUSG00000101939	16.55	-2.94	1.08E-04	*Gm28438*
ENSMUSG00000072844	528.14	-1.91	1.66E-04	*G530011O06Rik*
ENSMUSG00000089809	5939.70	-1.65	1.30E-84	*Rasgef1b*
ENSMUSG00000117465	58.16	-1.50	5.44E-05	*Gm49980*
ENSMUSG00000026580	56.68	-1.27	3.64E-02	*Selp*
ENSMUSG00000032827	37.90	-1.22	4.16E-02	*Ppp1r9a*
ENSMUSG00000041552	59.40	-1.15	9.94E-03	*Ptchd1*
ENSMUSG00000062345	347.62	-1.10	6.40E-09	*Serpinb2*
ENSMUSG00000000204	1329.80	-1.09	9.33E-25	*Slfn4*
ENSMUSG00000029084	222.04	-1.07	3.56E-06	*Cd38*
ENSMUSG00000050370	346.62	-1.02	6.24E-05	*Ch25h*
ENSMUSG00000028341	111.57	1.03	2.19E-04	*Nr4a3*
ENSMUSG00000047139	122.38	1.04	3.59E-04	*Cd24a*
ENSMUSG00000051457	125.50	1.06	8.93E-05	*Spn*
ENSMUSG00000041782	54.69	1.07	4.01E-02	*Lad1*
ENSMUSG00000028654	386.26	1.07	6.87E-11	*Mycl*
ENSMUSG00000034295	50.18	1.09	1.20E-02	*Fhod3*
ENSMUSG00000024538	135.21	1.15	1.94E-04	*Ppic*
ENSMUSG00000095041	1327.97	1.16	7.97E-27	*AC149090.1*
ENSMUSG00000019852	90.50	1.18	2.13E-04	*Arfgef3*
ENSMUSG00000000489	124.18	1.18	8.68E-06	*Pdgfb*
ENSMUSG00000004383	300.95	1.18	5.02E-05	*Large1*
ENSMUSG00000042842	329.08	1.21	2.03E-11	*Serpinb6b*
ENSMUSG00000045573	77.62	1.42	9.45E-05	*Penk*
ENSMUSG00000029223	215.04	1.48	5.07E-09	*Uchl1*
ENSMUSG00000021536	34.30	1.57	1.13E-02	*Adcy2*
ENSMUSG00000068735	50.69	1.60	7.49E-05	*Trp53i11*
ENSMUSG00000033174	21.40	1.72	3.04E-02	*Mgll*
ENSMUSG00000027864	26.31	1.75	8.70E-03	*Ptgfrn*
ENSMUSG00000052560	28.55	2.11	4.99E-04	*Cpne8*
ENSMUSG00000086695	17.58	2.14	4.38E-02	*Gm15247*
ENSMUSG00000025932	75.61	2.14	7.30E-10	*Eya1*
ENSMUSG00000039200	28.80	2.19	3.14E-05	*Atf7ip2*
ENSMUSG00000093954	17.28	7.51	1.07E-06	*Gm16867*
ENSMUSG00000045410	142.95	8.69	1.12E-13	*Akr1e1*

Table S5: List of RT-qPCR primers sequences

Gene	Direction	Sequence (5'- 3')
Rpl32	Forward	GCTGCCATCTGTTTTACGG
	Reverse	TGACTGGTGCCTGATGAACT
Rasgef1a	Forward	GTCACCCGTTGTGGACAAAG
	Reverse	TTGACCCGTTCCAGTTCAATG
Rasgef1b	Forward	GCTACAGCGAACGGAAGAA
	Reverse	CTTCACAGGCATGCAGATTTC
Rasgef1c	Forward	GCTGGAGCGACTAAGGCAC
	Reverse	CTGTTGAACCACTTCACGTAGG
Vcam1	Forward	AGTTGGGGATTCGGTTGTTCT
	Reverse	CCCCTCATTCCTTACCACCC
Ccl2	Forward	TTAAAAACCTGGATCGGAACCAA
	Reverse	GCATTAGCTTCAGATTTACGGGT
Ccl7	Forward	CCACATGCTGCTATGTCAAGA
	Reverse	ACACCGACTACTGGTGATCCT
Ccl8	Forward	TCTACGCAGTGCTTCTTTGCC
	Reverse	AAGGGGGATCTTCAGCTTTAGTA
Ccl12	Forward	ATTTCCACACTTCTATGCCTCCT
	Reverse	ATCCAGTATGGTCCTGAAGATCA
Cd38	Forward	TCTCTAGGAAAGCCCAGATCG
	Reverse	GTCCACACCAGGAGTGAGC
Selp1	Forward	CATCTGGTTCAGTGCTTTGATCT
	Reverse	ACCCGTGAGTTATTCCATGAGT
Ch25h	Forward	TGCTACAACGGTTCGGAGC
	Reverse	AGAAGCCCACGTAAGTGATGAT
Akr1e1	Forward	GCAGTTAAGTTGGCTATCAACCT
	Reverse	ACACCACAGCTTACTGACTACA
Serpinb2	Forward	ATTGGCAGTTATGGTATCACCAC
	Reverse	GGTGTGTTGATTGTTGAGCTGA
Slfn4	Forward	GGCTCCCTGCGTAAAGGAAC
	Reverse	GGGTAACATATTTTCGCGCTTG
Pkp2	Forward	GCCGAGTGTGGCTACATCC
	Reverse	CTGCTGGTTCGGTGAAGGTT
Pdgfb	Forward	CATCCGCTCCTTTGATGATCTT
	Reverse	GTGCTCGGGTCATGTTCAAGT
Eya1	Forward	TAACAGCTCGCCGTATCCAG
	Reverse	GTCCCAGATGAACACTCTCTCA
Ptchd1	Forward	ACACGGACCTGATCTTAAAGTTG
	Reverse	TTGGTGCCCATTATACACAGC
F13a1	Forward	GAGCAGTCCCGCCCAATAAC
	Reverse	CCCTCTGCGGACAATCAACTTA
